# Two Cases of Leiomyoma in the Colon Masquerading as Other Types of Colonic Pedunculated Polyps

**DOI:** 10.1155/2018/8272313

**Published:** 2018-04-29

**Authors:** Ailee Ikeda, Masaya Iwamuro, Takehiro Tanaka, Toshihiro Inokuchi, Asuka Nakarai, Yuusaku Sugihara, Keita Harada, Sakiko Hiraoka, Yoshiro Kawahara, Hiroyuki Okada

**Affiliations:** ^1^Department of Gastroenterology and Hepatology, Okayama University Graduate School of Medicine, Dentistry and Pharmaceutical Sciences, Okayama 700-8558, Japan; ^2^Department of Diagnostic Pathology, Okayama University Hospital, Okayama 700-8558, Japan; ^3^Department of General Medicine, Okayama University Graduate School of Medicine, Dentistry and Pharmaceutical Sciences, Okayama 700-8558, Japan; ^4^Department of Endoscopy, Okayama University Hospital, Okayama 700-8558, Japan

## Abstract

We describe two cases of leiomyoma in the colon that were diagnosed histologically after endoscopic resection. The first case was a 79-year-old Japanese woman who presented with a pedunculated polyp of 14 mm length at the splenic flexure. Preoperative diagnosis suggested a colonic mucosubmucosal elongated polyp. The second case was a 29-year-old Japanese woman who presented with a pedunculated polyp of 40 mm length at the hepatic flexure and had an ulcer on top of the polyp. Preoperative diagnosis suggested an inflammatory fibroid polyp. A pathological diagnosis of colonic leiomyoma was made after endoscopic resection in both cases. Both tumors were confirmed to originate, not from the proper muscle layer, but from the muscularis mucosae. These cases underscore that although colonic involvement is infrequent, leiomyomas can display pedunculated morphology in the colon rather than the typical gross appearance of gastrointestinal submucosal tumors seen with sessile morphology.

## 1. Introduction

Today, endoscopic examinations and interventions are extensively performed worldwide to investigate the digestive tract and to treat gastrointestinal diseases. Advances in endoscopic devices and techniques and in the understanding of typical endoscopic features of each disease enable an accurate preoperative diagnosis of various gastrointestinal lesions, even before performing the examination of biopsy samples. However, there are lesions that are not diagnosable by endoscopy.

In this report, we describe two cases of leiomyoma in the colon that were presented as pedunculated polyps. We could not diagnose either case as a leiomyoma preoperatively; the final diagnosis was made after endoscopic resection of the polyps. Differential diagnosis of these two cases and morphology of the colorectal leiomyomas are discussed.

## 2. Case Report

### 2.1. Case 1

**Figure 1:**
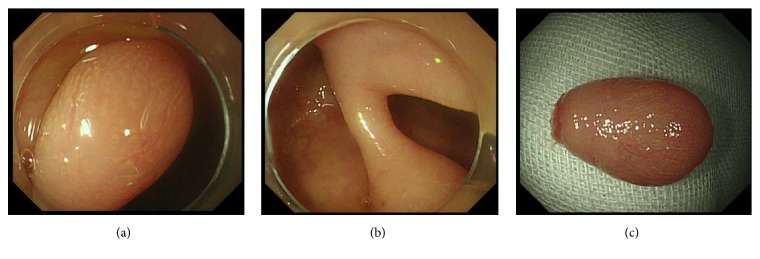
Endoscopic images of Case 1. A pedunculated polyp covered with intact mucosa is observed (a, b). Resected polyp is 10 mm in diameter and 14 mm in length (c).

**Figure 2:**
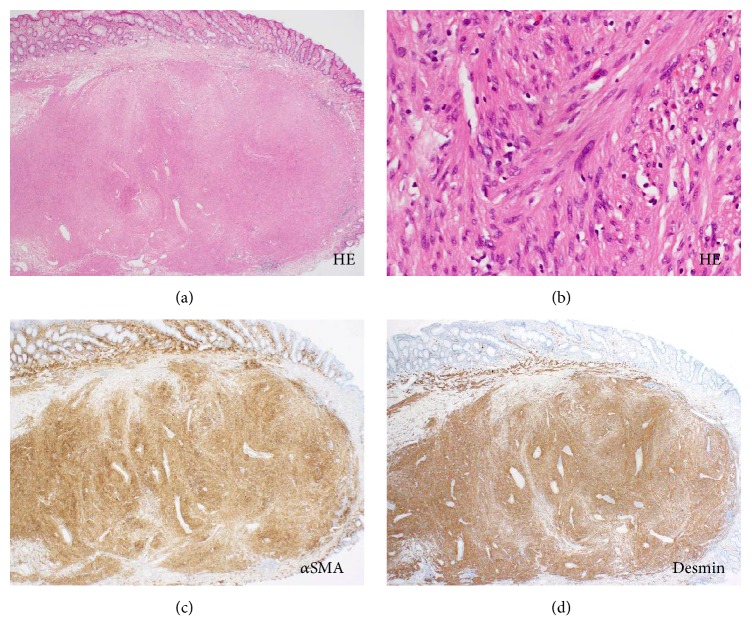
Pathological images of Case 1. A tumor composed of eosinophilic cells is observed. The tumor is covered with normal appearing mucosa ((a), hematoxylin and eosin staining, ×40). The tumor cells have relatively uniform nuclei, abundant cytoplasm, and indistinct cell boundaries. Mitoses are rarely observed ((b), hematoxylin and eosin staining, ×400). The cells are positive for *α*-SMA ((c), ×40) and desmin ((d), ×40).

A 79-year-old Japanese woman was referred to our hospital for further investigation of a positive fecal occult blood test. A blood examination revealed no specific abnormalities. Colonoscopy revealed an oval-shaped pedunculated polyp at the splenic flexure of the transverse colon (Figure 1). The surface of the polyp was covered with intact mucosa; regularly arranged round pits were observed under magnification (Figure 1(a)). A preoperative diagnosis of colonic mucosubmucosal elongated polyp was made, and an endoscopic submucosal dissection was performed on the basis of this diagnosis. The resected polyp was 10 mm in diameter and 14 mm in length (Figure 1(c)). Histologically, dense proliferation of spindle cells was observed in the submucosal layer (Figures 2(a) and 2(b)). Tumor nests were clearly demarcated from the overlying mucosa by fibrous connective tissue. Continuity between the tumor and the muscularis mucosae was observed, indicating that the tumor arose from the muscularis mucosae. Mitoses were rarely observed. The cells were positive for *α*-smooth muscle cell actin (*α*-SMA, Figure 2(c)) and desmin staining (Figure 2(d)) but were negative for c-kit, S-100, or DOG-1 staining. Consequently, a diagnosis of colonic leiomyoma was made.

### 2.2. Case 2

**Figure 3:**
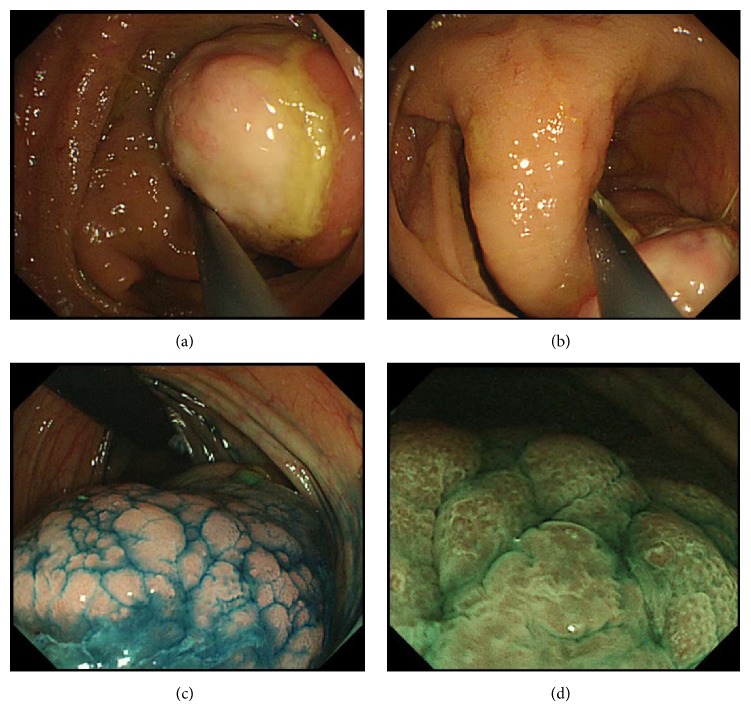
Endoscopic images of Case 2. The surface of the polyp head is ulcerated (a). The polyp is of pedunculated form (b). The unulcerated part of the polyp shows intact pit structure after indigo carmine spraying (c). Narrow-band imaging (d).

**Figure 4:**
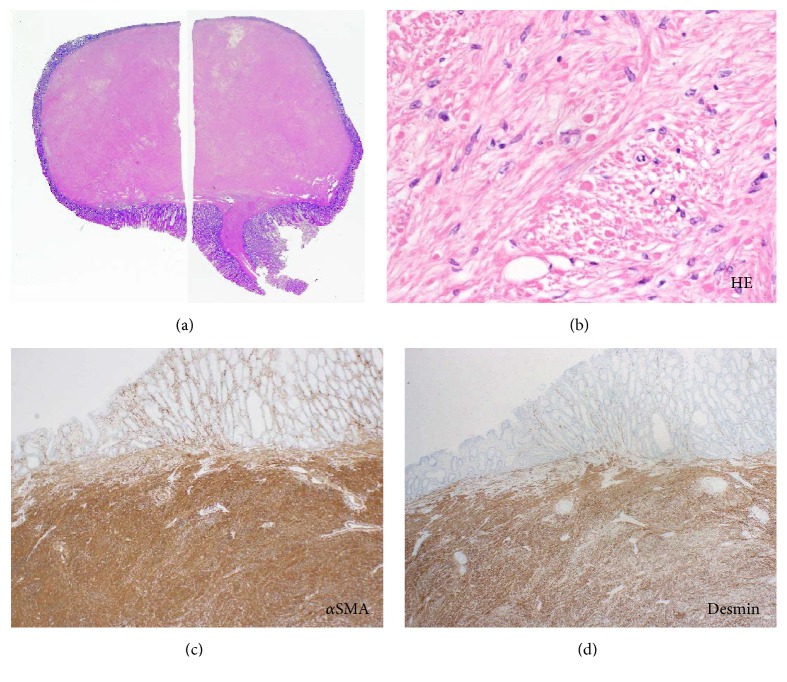
Pathological images of Case 2. Spindle cells with round to oval nuclei are observed in the resected specimen ((a), ×4, (b), ×400). The tumor cells are positive for *α*-SMA ((c), ×100) and desmin ((d), ×100).

A 29-year-old Japanese woman was referred to our hospital for further investigation of abdominal pain and bloody stools. Laboratory investigation revealed microcytic hypochromic anemia with red blood cell count: 3.92 × 10^6^/mm^3^, hemoglobin: 8.5 g/dL, hematocrit: 29.4%, platelet count: 373 × 10^3^/mm^3^, and C-reactive protein: 0.53 mg/dL. A pedunculated polyp, 40 mm in diameter and 40 mm in length, was identified at the hepatic flexure of the colon during colonoscopy (Figures 3(a) and 3(b)). The surface of the polyp was ulcerated and covered with a white substance (Figure 3(a)). Although the mucosa of the unulcerated parts of the polyp was partly swollen, the pit structure was mostly normal and round; this was emphasized after indigo carmine spraying (Figure 3(c)). Narrow-band imaging, under magnification, also showed partial swelling of the surface structure (Figure 3(d)). Based on the pedunculated morphology and partial ulceration, our preoperative diagnosis of the polyp suggested an inflammatory fibroid polyp. We removed the polyp using the endoscopic mucosal resection technique. Histological analysis revealed a dense proliferation of spindle cells with round to oval nuclei beneath the thin, normal mucosal epithelium (Figures 4(a) and 4(b)). Mitoses were rarely observed. The stalk of the polyp was composed of muscular tissue. Tumor cells were positive for *α*-SMA (Figure 4(c)) and desmin (Figure 4(d)) but were negative for c-kit, S-100, or DOG-1. Therefore, we made a pathological diagnosis of leiomyoma.

## 3. Discussion

According to Japanese clinical practice guidelines for colonic polyps, they are classified into at least eight types: conventional adenomas, serrated polyps, polypoid adenocarcinomas, inflammatory polyps, hamartomatous polyps, stromal polyps, lymphoid polyp, and endocrine (or carcinoid) tumors, as well as others [1]. Although lesions arising from or involving colon mucosa can be easily identified and diagnosed during colonoscopy, based on the morphological structure of the mucosal surface, it is generally difficult to immediately diagnose subepithelial lesions covered with normal mucosa. The two cases under study had pedunculated polyps that we diagnosed as a colonic mucosubmucosal elongated polyp and an inflammatory fibroid polyp, preoperatively.

As pertains to the gastrointestinal tract, leiomyomas occur most frequently in the esophagus while the colorectum is a relatively infrequent site of occurrence, accounting for 3% of all the gastrointestinal leiomyomas [2–5]. Since a majority of esophageal leiomyomas originate from the proper muscle layer, they present as sessile lesions covered with intact esophageal mucosa, that is, the typical appearance of subepithelial tumors. Meanwhile, it has been reported that colonic leiomyomas arise from the muscularis mucosae. Although colonic leiomyomas predominantly appear as sessile lesions, they can form semipedunculated or pedunculated lesions as well [6]. Choi et al. reported 22 cases with leiomyomas arising from the muscularis mucosae in the colon [7]. Seventeen cases showed sessile polyps, while the remaining five cases showed pedunculated morphology. Agaimy and Wünsch reported that all of the 67 colorectal leiomyomas arising from the muscularis mucosae were well-circumscribed, sessile polypoid masses detected incidentally during colonoscopy [6]. Miettinen et al. reviewed 88 patients with leiomyoma of the muscularis mucosae and described that the lesions typically appeared as small sessile polyps [8]. Hence, it is evident that colonic leiomyomas present semipedunculated or pedunculated lesions only in rare instances.

Colonic leiomyomas are often misdiagnosed. Several authors have noted that most colonic leiomyomas with sessile morphology mimic epithelial polyps in their gross appearance during colonoscopy [4, 7]. Choi et al. reported that 45.5% of colorectal leiomyomas (10 of 22 lesions) were accurately diagnosed based on their endoscopic features [7]. However, more than half of colorectal leiomyomas were not correctly diagnosed prior to undergoing resection. We speculate that it is more difficult for gastroenterologists to diagnose pedunculated lesions as leiomyomas preoperatively because of their infrequency and physician's prejudices that leiomyomas form sessile lesions and not pedunculated lesions. In the two cases under study, our preoperative diagnosis for one case was mucosubmucosal elongated polyp and, for the other, inflammatory fibroid polyp.

Colonic mucosubmucosal elongated polyp is a distinct entity that is composed of mucosa and submucosa [9]. Typical endoscopic features have been identified as worm-like, thin projections covered by unremarkable mucosa [10]. Our patient, from Case 1, showed a pedunculated polyp lined with intact mucosa. Although subepithelial tumors rarely display pedunculated morphology, we have considered subepithelial tumors as a differential diagnosis of the polyp identified in Case 1 because the polyp head seemed to be thicker than a typical colonic mucosubmucosal elongated polyp.

The inflammatory fibroid polyp is a rare mesenchymal gastrointestinal tumor found mostly in the stomach (70%) and the small intestine (20%) [11]. Involvement of the colon in this disease is infrequent. However, reports of inflammatory fibroid polyp of the proximal colon, particularly those of the cecum, have increased due to recent advances in and widespread use of colonoscopy examinations [12]. Pathologically, an inflammatory fibroid polyp is characterized by blood vessels, fibroblasts, and an edematous stroma rich in eosinophils [13]. Representative endoscopic features of this entity are a firm, solitary, and often ulcerated polyp with sessile or pedunculated form [14]. We consider that colonic inflammatory fibroid polyp is a reasonable preoperative diagnosis for the patient in Case 2, as it is hard to conceive of the polyp as a leiomyoma.

Unlike the esophageal leiomyoma, colonic leiomyomas can be endoscopically removed because most of them arise from the muscularis mucosa. Choi et al. report that they have removed colorectal leiomyomas using cold forceps biopsy (*N* = 10) and conventional polypectomy or endoscopic mucosal resection (*N* = 12) and have experienced no complications such as bleeding or perforation [7]. Miettinen et al. summarized 88 colorectal leiomyomas and noted that endoscopic snare polypectomy resulted in complete tumor removal in virtually all cases [8]. The overall prognosis is reportedly good, and no recurrence has been documented in literature [15]. Thus, endoscopic resection is a treatment of choice for leiomyomas arising from the muscularis mucosa of the colon. In the two cases under discussion, we could not preoperatively diagnose the polyps as leiomyomas; a final diagnosis was made after endoscopic resection. In such cases, additional treatment is unnecessary, if the leiomyoma arises from the muscularis mucosa.

In conclusion, we treated two cases of colonic leiomyoma presented as pedunculated polyps. Accurate preoperative diagnoses were difficult based solely on their morphology. These cases underscore that although colonic involvement is infrequent, colonic leiomyomas can display the pedunculated form, rather than the typical gross appearance of gastrointestinal submucosal tumors as in the sessile form. In addition, they can be treated with endoscopic resection alone because most of colonic leiomyomas arise from the muscularis mucosa.
